# Fulminant Pyopericardium: A Case Report

**DOI:** 10.7759/cureus.73668

**Published:** 2024-11-14

**Authors:** Teresa Costa e Silva, Inês Ferreira Neves, Luís Almeida Morais, Inês Rodrigues, Rui Cruz Ferreira

**Affiliations:** 1 Internal Medicine, Hospital Beatriz Ângelo, Unidade Local de Saúde Loures-Odivelas, Loures, PRT; 2 Cardiology, Hospital de Santa Marta, Unidade Local de Saúde São José, Lisbon, PRT

**Keywords:** acute endocarditis, methicillin-sensitive staphylococcus aureus (mssa), pyopericardium, septic shock, transesophageal echocardiogram

## Abstract

Pyopericardium has been seldom reported in the literature and carries a high mortality. The authors present the case of a 67-year-old woman with a pyopericardium diagnosis, masked by chest pain related to subacute, non-revascularized coronary syndrome. We report this case not only due to its uniqueness but also to raise awareness of the importance of a rapid diagnostic and treatment approach.

## Introduction

Pyopericardium is a clinical rarity in which a pus-filled pericardial effusion is present. Its pathogenesis is allied to the fact that virtually any organism can spread to the pericardial sac. There are different mechanisms by which pyopericardium may develop, namely: direct spread, such as contiguous extension from intrathoracic infections; via the hematogenous route, as occurs in septicemia; or by extension from a suppurative focus, as in subdiaphragmatic infections [1]. Especially in cases with hematogenous sources, the most commonly implicated pathogens are *Staphylococcus aureus* and streptococcal species [2,3].

Pyopericardium etiology has evolved from predominantly infectious causes to non-infectious ones, including malignancies, chronic renal failure, and postoperative complications, making cases with an infectious origin, such as the one the authors present, increasingly rare [1].

Presenting symptoms and signs often include chest pain, high fever, and signs of hemodynamic instability, resembling acute coronary or inflammatory cardiac conditions, which may delay diagnosis [4]. Therefore, its diagnosis remains a significant clinical challenge, with high mortality rates associated if not rapidly managed. Prompt diagnosis, followed by aggressive treatment with pericardial drainage and systemic antimicrobials, is crucial for decreasing mortality [1].

This rare case illustrates how challenging the diagnostic and therapeutic approach to a fulminant pyopericardium can be, and highlights the intricate interplay between septic dissemination and cardiac pathology.

## Case presentation

The authors present the clinical case of a 67-year-old woman with a history of uncontrolled type 2 diabetes mellitus, arterial hypertension, and obesity, who was admitted to the emergency department with retrosternal pain, nausea, vomiting, and hyperglycemia over two days. Her physical examination revealed a systolic blood pressure of 70 mmHg and diminished breath sounds in the lower right lung field.

**Figure 1:**
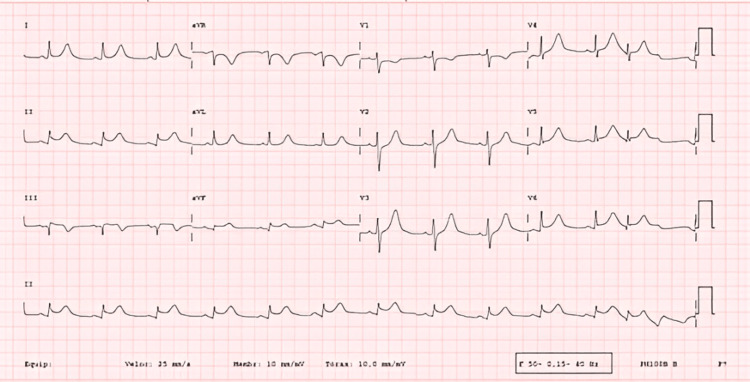
Complementary study on admission - electrocardiogram. Electrocardiogram showing Q waves and ST segment elevation in DII, DIII, and augmented vector foot (aVF) leads.

An electrocardiogram was performed, which showed sinus rhythm, as well as a new onset of Q waves and ST-segment elevation in the DII, DIII, and augmented vector foot (aVF) leads (Figure 1).

The diagnosis of acute ST-elevation myocardial infarction (STEMI) was presumed, and aspirin, ticagrelor, and low-molecular-weight heparin (LMWH) were administered. The patient was then transferred to our percutaneous coronary intervention (PCI) center for emergent cardiac catheterization.

**Figure 2:**
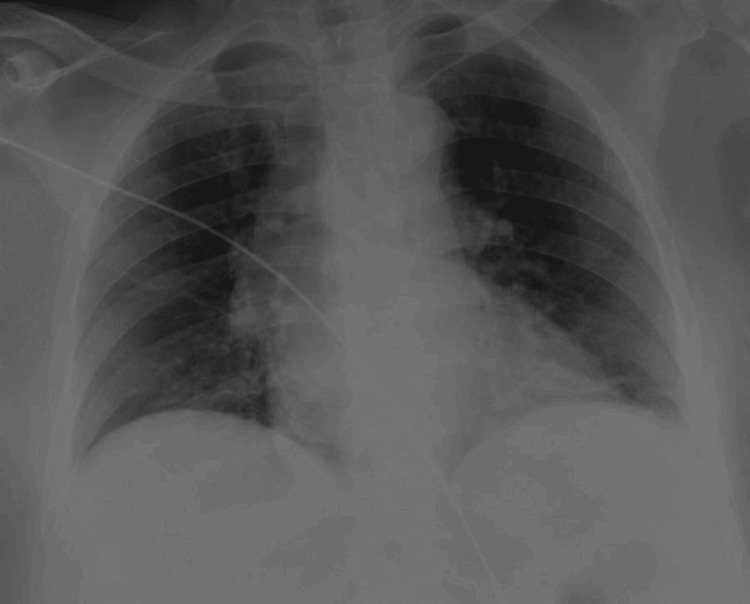
Complementary study on admission - anteroposterior chest radiography. Anteroposterior chest radiography shows cephalic vascularization and scattered broncho-vascular reinforcement, with no water bottle sign present.

On admission, laboratory workup demonstrated subtle anemia, slight leukocytosis, thrombocytopenia, augmented values of D-dimer, creatine kinase, and troponin T, and marked elevation of C-reactive protein (Table 1); anteroposterior chest radiography exhibited cephalic vascularization and scattered broncho-vascular reinforcement (Figure 2); and point-of-care echocardiography disclosed a non-dilated right and left ventricle with preserved biventricular ejection fraction, hypokinesia of the mid-basal inferior wall, no signs of significant valvulopathies, and absence of pericardial effusion.

**Table 1:** Complementary study on admission - laboratory workup.

Laboratory exams	Laboratory findings	Reference levels
Hemoglobin	11.4 g/dL	12.0-15.0 g/dL
Mean corpuscular volume	75.4 fL	78.0-96.0 fL
Mean corpuscular hemoglobin	24.80 pg	26.0-33.0 pg
White blood cells	10.6 x 10^9^/L	4.0-10.0 x 10^9^/L
Platelets	120.0 x 10^9^/L	150.0-450.0 x 10^9^/L
D-dimer	4325.0 µg/L	<500.0 µg/L
Creatine kinase	345.0 UI/L	<170.0 UI/L
Troponin T	443.0 ng/L	<14.0 ng/L
C-reactive protein	250.9 mg/L	<5.0 mg/L

**Figure 3:**
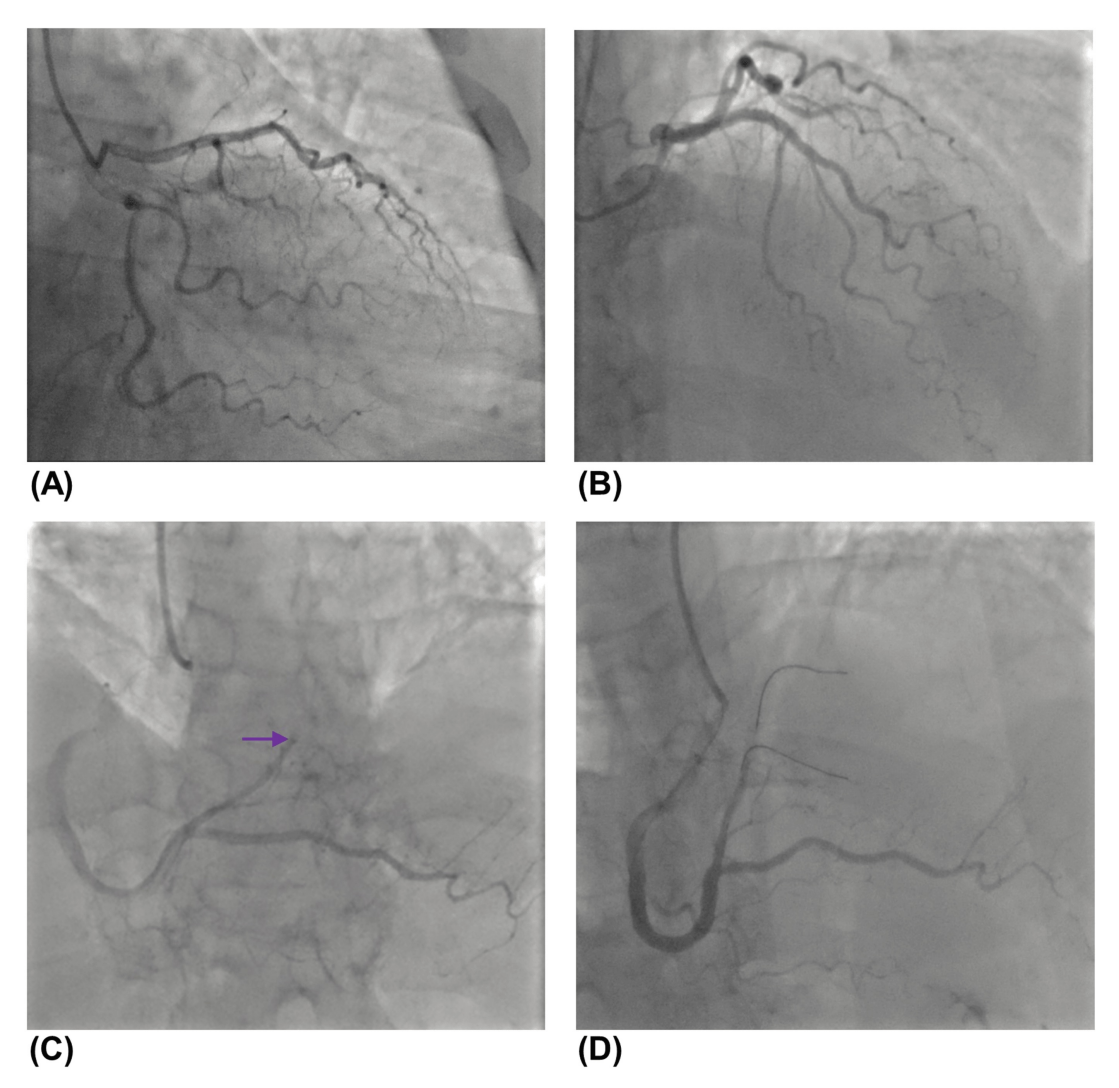
Complementary study on admission - cardiac catheterization. Cardiac catheterization showing the left coronary system with no obstruction (A), right coronary artery (B) with occlusion (purple arrow) of the distal right posterolateral branch (PLB) (C), and angioplasty of PLB unsuccessfully performed (D).

Cardiac catheterization revealed single-vessel disease, with right first posterolateral branch distal occlusion. PCI was attempted; nevertheless, it was unsuccessful, attributable to the small caliber of the vessel and the lesion’s location (Figure 3).

The patient was then admitted to our cardiac intensive care unit (ICU). Despite the absence of new electrocardiographic alterations and a decrease in troponin (234 ng/L), she continued to experience chest pain and developed a fever, leading to suspicion of respiratory infection, obtainment of cultures, and initiation of empiric antibiotic therapy with amoxicillin and azithromycin.

Approximately 10 hours after admission to the ICU, the patient developed new-onset atrial fibrillation with a rapid ventricular rate, accompanied by signs of hemodynamic instability and respiratory distress, requiring electrical cardioversion. Given the clinical worsening with increased hyperlactatemia, evolving into shock, vasopressor support, invasive mechanical ventilation, and corticotherapy were needed.

**Figure 4:**
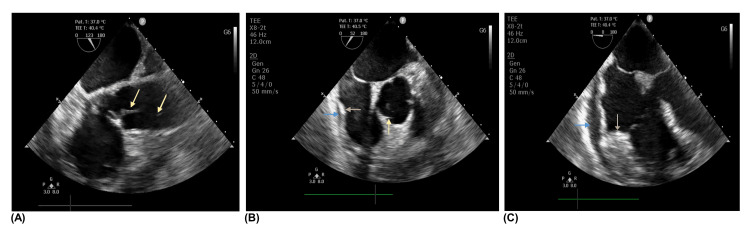
Complementary study during ICU stay - transesophageal echocardiogram. Transesophageal echocardiogram showing large aortic valve vegetation (yellow arrows) (A), moderate pericardial effusion (blue arrows), and echogenic image of moderate dimensions in right atrium-ventricle junction (grey arrows) (B-C). ICU, intensive care unit

A point-of-care echocardiogram was performed to better comprehend the type and cause of the shock and to help rule out mechanical complications. Despite a poor echocardiogram view, a new finding was revealed: moderate pericardial effusion, leading to right atrium retraction. A transesophageal echocardiogram (TEE) uncovered a filiform image with hypermobility and considerable dimensions affixed to the aortic valve, an echogenic image of moderate dimensions at the right atrium-ventricle junction, both suggestive of abscesses, and moderate to severe pericardial effusion (Figure 4). Neither aortic valve insufficiency nor tamponade physiology nor right chamber collapse were present, which favored the consideration of septic shock as the diagnosis.

**Figure 5:**
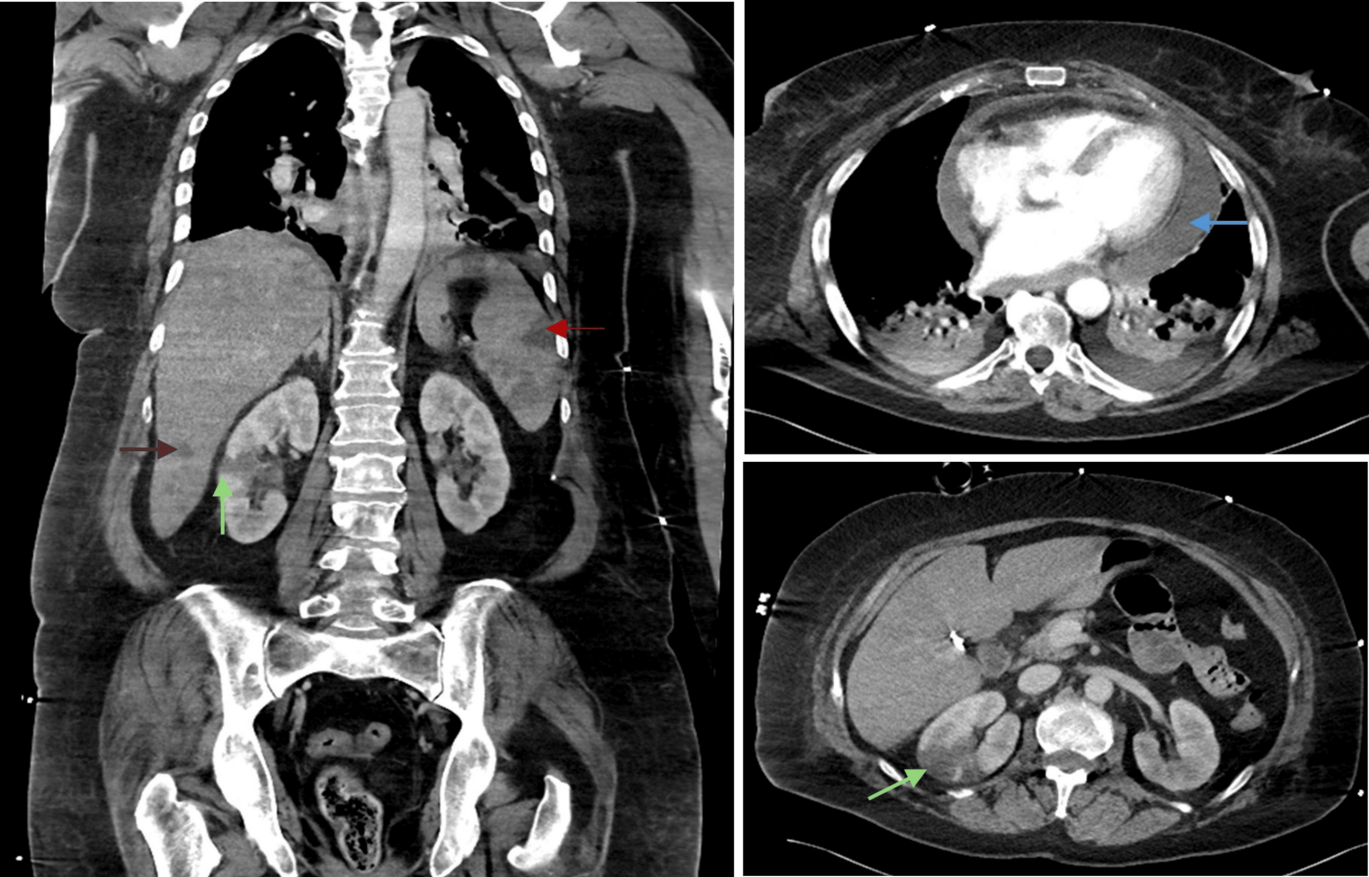
Complementary study during ICU stay - thoraco-abdominopelvic CT scan with angiography. Thoraco-abdominopelvic CT scan with angiography revealing high density pericardial effusion on the upper right (blue arrow), multiple renal (green arrows) and splenic (red arrow) infarction, and hepatic abscesses (brown arrow). ICU, intensive care unit; CT, computed tomography

A thoraco-abdominopelvic computed tomography (CT) angiography scan, performed to exclude aortic dissection, documented the pericardial effusion as being 23 mm thick and high density, along with multiple renal and splenic infarctions, hepatic abscesses, and no signs of aortic dissection (Figure 5). With the diagnosis of septic shock due to severe infective endocarditis with pericardial effusion and possible bilateral cardiac abscesses, empiric loading doses of vancomycin and gentamicin were administered. Blood cultures were obtained during clinical deterioration, which isolated methicillin-sensitive *Staphylococcus aureus* (MSSA), allowing for targeted antibiotic therapy.

**Figure 6:**
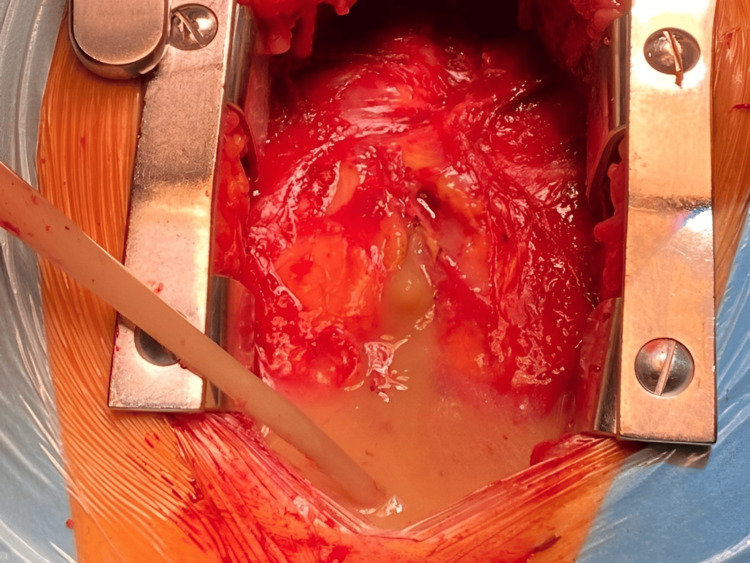
Pericardiotomy displaying unequivocal pyopericardium.

Gathering all the findings, emergent cardiac surgery was performed. Intraoperatively, a massive pyopericardium was noted (Figure 6); furthermore, perivalvular aortic thickening and macroscopic, dispersed, whitened infiltration of the myocardium were observed. Nevertheless, no evidence of vegetation on the aortic valve or myocardial infarction scar was found. An aortic valve replacement was performed, along with surgical drainage and pericardiotomy. The pericardial fluid volume, turbid appearance, and cytology, presenting abundant polymorphonuclear cells, were consistent with an exudate, as suspected. Both pericardial fluid culture and aortic valve sample revealed MSSA.

Despite the prompt diagnosis and rapid treatment attempt, the patient progressed to refractory septic shock with multi-organ dysfunction approximately 38 hours after admission to the ICU, leading to death.

## Discussion

Pyopericardium, a clinical rarity characterized by pus accumulation in the pericardial space, carries a mortality risk of 100% if left untreated, which decreases to 40% with treatment [4].

Historically linked to infectious diseases, non-infectious predisposing conditions are now more frequently seen [5]. This evidence supports the uniqueness of our case, as the patient presented with an underlying infectious disease, which is less common in the antibiotic era.

A pyopericardium may develop through various mechanisms, namely, direct spread from intrathoracic infections, the hematogenous route, or extension from subdiaphragmatic suppurative foci [5]. Less frequently, cases of myocardial infarction complicated by infection, resulting in abscess formation and secondary pyopericardium, have been described. Myocardial abscesses can also arise as metastatic infections in patients with bacteremia due to organisms such as *S. aureus* or *Salmonella* [6]. Rarely, coronary artery stents can become infected with *S. aureus*, resulting in aneurysms and pyopericardium [7]. The most common pathogens implicated in hematogenous spread are *S. aureus* and strains from the *Streptococcaceae* family.

A few aspects of our case are noteworthy regarding etiology. First, our patient had bacteremia due to *S. aureus*, which we initially believed to be of respiratory origin. However, during the diagnostic approach, no signs of infection were demonstrated on the CT scan, and TEE findings were more prone to acute endocarditis diagnosis.

Second, she was admitted due to STEMI, underwent cardiac catheterization, and showed no signs of pericardial effusion on the initial point-of-care echocardiogram or on the admission chest radiography. Presumably, she developed a moderate pericardial effusion within approximately 24 hours. On the contrary, we believe her clinical context may have begun much earlier than the patient reported.

Third, a suspicion of aortic valve abscess was also acknowledged, and hepatic abscesses of unknown origin were detected on the CT scan.

The authors consider several characteristics that could match all the described mechanisms of pyopericardium formation; nevertheless, we believe that both the direct extension from acute endocarditis and the hematogenous route may better explain the formation of pyopericardium. While we cannot be completely sure of the exact route of pyopericardium formation, the significant bacteremia and focal embolization favor this as a septicemia-related case of pancarditis.

Most patients with pyopericardium are acutely ill. Symptoms such as high fever, tachycardia, and chest pain are commonly reported [5]. She exhibited these symptoms and rapidly progressed to shock, which warned us of her critical condition in the means of the rapid atrial fibrillation triggered by the high sympathetic state she was in.

Prompt diagnosis, established by obtaining pericardial fluid for culture and microscopy, followed by aggressive treatment with pericardial drainage and systemic antibiotics, is crucial for decreasing mortality [8]. Nevertheless, its diagnosis is frequently delayed due to the initial source of infection distracting from the underlying cardiac problem, and the common reporting of the same symptoms as acute coronary syndrome, which led to our patient’s ICU admission. In this context, the authors would like to note that the electrocardiogram provided could raise a clinical hypothesis of acute pericarditis. However, given the patient’s history of various cardiovascular risk factors, oppressive chest pain, and elevated troponin levels upon admission, we deemed it important to rule out acute coronary syndrome by performing emergent cardiac catheterization, as the presence of this diagnosis could change the course of treatment and impact the patient’s prognosis. During the procedure, the authors noted that the infarct-related artery appearance was suggestive of a chronic occlusion rather than an embolic lesion, supported by unsuccessful PCI.

No consensus exists on the optimal management of pyopericardium. Considering the pericardial effusion’s characteristics and the fact that the TEE did not exclusively demonstrate abnormalities of the pericardium, the authors believe that the pericardiotomy performed was the best drainage approach for the referred patient. Given the TEE and intraoperative findings, the presence of such a severe infectious condition, and the inflammatory insult associated with the cardiopulmonary bypass already performed, it was a challenging decision; however, the authors decided to proceed with an aortic valve replacement.

Intravenous antibiotics should be initiated promptly upon suspicion [8]. We should bear in mind that the patient was submitted to cardiac catheterization; however, this intervention was performed with high standards of asepsis. Furthermore, the hemoculture, pericardial effusion, and aortic valve results came out earlier than usual, possibly due to the high bacterial load in the bloodstream. Antibiotic therapy directed at *S. aureus* was prescribed as soon as the results were found; however, this life-threatening condition imposed itself.

## Conclusions

This case highlights the prevailing, unfortunate outcome of pyopericardium and intends to create greater awareness of the disease and its diagnostic and treatment approach. The patient’s clinical deterioration was extremely fast, despite our attempts to prevent her death, supporting the importance of timely recognition and treatment of pyopericardium as paramount for better outcomes.

## References

[1] Parikh SV, Memon N, Echols M, Shah J, McGuire DK, Keeley EC (2009). Purulent pericarditis: report of 2 cases and review of the literature. Medicine (Baltimore).

[2] Nwiloh JO, Egbe PA, Tagoe AT, Weaver LW (2000). Staphylococcus aureus pericarditis masquerading as anterior mediastinal mass: mediastinal mass from pericarditis. Chest.

[3] Rubin RH, Moellering RC Jr (1975). Clinical, microbiologic and therapeutic aspects of purulent pericarditis. Am J Med.

[4] Cracknell BR, Ail D (2015). The unmasking of a pyopericardium. BMJ Case Rep.

[5] Sagristà-Sauleda J, Barrabés JA, Permanyer-Miralda G, Soler-Soler J (1993). Purulent pericarditis: review of a 20-year experience in a general hospital. J Am Coll Cardiol.

[6] Katz A (1964). Abscess of the myocardium complicating infarction: report of two cases. Can Med Assoc J.

[7] Schoenkerman AB, Lundstrom RJ (2009). Coronary stent infections: a case series. Catheter Cardiovasc Interv.

[8] Adler Y, Charron P, Imazio M (2015). 2015 ESC guidelines for the diagnosis and management of pericardial diseases: the task force for the diagnosis and management of pericardial diseases of the European Society of Cardiology (ESC) endorsed by: the European Association for Cardio-Thoracic Surgery (EACTS). Eur Heart J.

